# Disinfection of Respirators with Ultraviolet Radiation

**DOI:** 10.6028/jres.126.058

**Published:** 2022-03-25

**Authors:** Dianne L. Poster, Matthew Hardwick, C. Cameron Miller, Michael A. Riley, W. W. Shanaka I. Rodrigo, Andras E. Vladar, John D. Wright, Christopher D. Zangmeister, Clarence Zarobila, Jeremy Starkweather, John Wynne, Jason Yilzarde

**Affiliations:** 1 National Institute of Standards and Technology, Gaithersburg, MD 20899, USA; 2 ResInnova Laboratories, Rockville, MD 20857, USA; 3 UV-Concepts, Inc., Englewood, CO 80112, USA

**Keywords:** disinfection, efficacy, face masks, flow resistance, gas flow, microscopy, N95, particle filtration, personal protective equipment, PPE, respirators, scanning electron microscopy, SEM, tensile strength, ultraviolet-C, ultraviolet germicidal irradiation, UV-C, UVGI, virus

## Abstract

Data for interpreting virus inactivation on N95 face filtering respirators (FFRs) by ultraviolet (UV) radiation are important in developing UV strategies for N95 FFR disinfection and reuse for any situation, whether it be everyday practices, contingency planning for expected shortages, or crisis planning for known shortages. Data regarding the integrity, form, fit, and function of N95 FFR materials following UV radiation exposure are equally important. This article provides these data for N95 FFRs following UV-C irradiation (200 nm to 280 nm) in a commercial UV-C enclosure. Viral inactivation was determined by examining the inactivation of OC43, a betacoronavirus, inoculated on N95 FFRs. Different metrological approaches were used to examine irradiated N95 FFRs to determine if there were any discernible physical differences between non-irradiated N95 FFRs and those irradiated using the UV-C enclosure. Material integrity was examined using high-resolution scanning electron microscopy. Form, fit, and function were examined using flow resistance, tensile strength, and particle filtration measurements. A separate examination of filter efficiency, fit, and strap tensile stress measurements was performed by the National Personal Protective Technology Laboratory. Data from these metrological examinations provide evidence that N95 FFR disinfection and reuse using the UV-C enclosure can be effective.

## 1 Introduction

Potential shortages of personal protective equipment (PPE) are a problem for personal and public safety in an emerging or sustained health pandemic,^1^1As reviewed by Singer *et al.* [1], “the *International Epidemiology Association’s Dictionary of Epidemiology* defines a pandemic as ‘an epidemic occurring worldwide, or over a very wide area, crossing international boundaries and usually affecting a large number of people.’” or a natural disaster, such as a catastrophic hurricane, flood, or wildfire. Respirators are one component of a suite of PPE that offers protection for front- and second-line responders, healthcare personnel and patients in such conditions. Respirators reduce the risk of inhaling hazardous airborne particles, including small particle aerosols, large droplets, and biological pathogens.

**Fig. 1:**
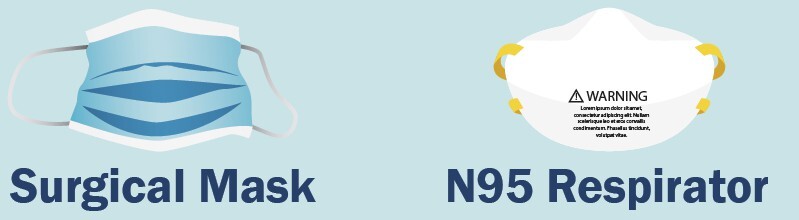
Graphic showing the difference between a facemask (sometimes called a surgical mask) and N95 filtering facepiece respirator (N95 FFR, sometimes called N95 respirators). Surgical masks are cleared by the U.S. Food and Drug Administration. N95 FFRs are evaluated, tested, and approved by NIOSH as per the requirements in 42 Code of Federal Regulation (CFR) Part 84^3^3U.S. Code of Federal Regulations (1995) Title 42 CFR Part 84—Approval of Respiratory Protective Devices. https://www.ecfr.gov/current/title-42/chapter-I/subchapter-G/part-84. Surgical masks are loose fitting and do not require fit testing. N95 FFRs are tight fitting and require fit testing. Surgical masks offer fluid resistance and provide the wearer protection against large droplets, splashes, or sprays of bodily or other hazardous fluids. In addition, surgical masks protect the patient from the wearer’s respiratory emissions. N95 FFRs reduce a wearer’s exposure to particles including small particle aerosols and large droplets (only non-oil aerosols). Graphic and information are from Ref. [2], where more information on PPE is available.

Filtering facepiece respirators (FFRs) remove particles from the air as it is breathed inward by the user. FFRs with the N95^2^2“N95” is a filter class designation of the U.S. National Institute of Occupational Safety and Health (NIOSH). It is applied to respirators that are at least 95% efficient at filtering NaCl aerosols with particle sizes of mean diameter 75 nm ± 20 nm (NIOSH Procedure No. TEB-APR-STP0059, 13 December 2019). See Ref. [2] for the U.S. Centers for Disease Control and Prevention definition of an N95 filtering facepiece respirator (N95 FFR) and Fig. 1. designation remove at least 95% of small (0.3 μm) particles and filter all types of particles, including bacteria and viruses [2]. N95 FFRs are not the same as facemasks (sometimes called surgical masks) (Fig. 1) [2]. This article is focused on the disinfection and reuse of N95 FFRs.

Ultraviolet-C radiation (UV-C)^4^4The UV-C range (200 nm to 280 nm) is sometimes called the germicidal range because it is effective for inactivating bacteria and viruses. See Bolton and Cotton [3] for more information on UV light spectral ranges and emission, transmission, and absorption of light, noting “physicists prefer to use the term [UV] radiation, reserving the term light for visible light” [3]. Similarly, UV radiation is the term preferred at the National Institute of Standards and Technology (NIST) to describe the energy emitted in the UV portion of the electromagnetic spectrum (100 nm to 400 nm) [4]. has been shown to be effective for decontaminating FFRs contaminated with influenza, even with the presence of soiling agents on the FFRs like artificial saliva or artificial skin oil [5]. Significant reductions in influenza viability (≥ 3 log_10_ reductions^5^53 log_10_ units typically refers to a 99.9% reduction, calculated as log_10_ (*N_0_*/*N*), where *N_0_* is the initial viable microorganism count, and *N* is the final value after UV exposure (see Masjoudi *et al.* [6] for a compilation of recommended fluence values [UV dose] for specific log reductions of bacteria, protozoa, viruses, and other microorganisms).) were achieved at a UV dose^6^6Kumar *et al*. [7] recently noted that “it is recognized microbial pathogens are susceptible to a range of cumulative UV-C dosing, usually measured in mJ/cm^2^. Commercially available UV-C devices emit at a variety of UV-C light intensity, usually measured in mW/cm^2^. The weaker the intensity, the greater time required to reach the desired cumulative dose.” With that said, Kumar *et al*. found that the required UV-C cumulative dose for reductions of *Staphylococcus aureus* on carriers is independent of applied UV-C intensity [7]. of 1 J/cm^2^ for 12 of 15 UV-treated FFR models [5]. The remaining three models still demonstrated statistically significant reductions in virus viability, but it was concluded that additional research was required to define how specific FFR design attributes, such as FFR materials, design, and shape, may influence UV efficiency [5]. Regardless, this study provided critically important data to understand UV-based FFR disinfection and reuse strategies in the event of a viral pandemic.

Due to the outbreak of severe acute respiratory syndrome coronavirus 2 (SARS-CoV-2), the virus that causes coronavirus disease 2019 (COVID-19), FFR disinfection and reuse strategies for healthcare providers gained attention. In February 2020, the U.S. Department of Health and Human Services testified to the U.S. Congress that federal stockpiles required 300 million N95 respirator masks to adequately respond to the outbreak [8]. Because of shortages, reuse of PPE was reportedly widespread [8] and strategies for optimizing the supply of N95 FFRs were formulated by the U.S. Centers for Disease Control and Prevention (CDC) [9]. The reuse of one mask, one time, offered the ability to cut the demand for N95 respirator masks in half at any given location.

By April 2020, extreme shortages of disposable FFRs were predicted, and the use of UV-C to disinfect masks of SARS-CoV-2 was recommended by the medical community [10]. The U.S. National Institute for Occupational Safety and Health (NIOSH) found that, as of April 2020, UV germicidal irradiation, vaporous hydrogen peroxide, and moist heat were the most promising potential methods with which to decontaminate^7^7Decontamination is a process to reduce the number of pathogens on used FFRs before reusing them [9]. Disinfection describes a process that eliminates many or all pathogenic microorganisms, except bacterial spores, on inanimate objects [11]. FFRs [9]. As reviewed by Cassorla [12], there was a surge of internet-available information and guidance on the decontamination and reuse of N95 FFRs from the CDC, the U.S. Food and Drug Administration, the Emergency Care Research Institute, a nonprofit health quality research institute, N95decon.org, a volunteer consortium dedicated to N95 FFR decontamination and reuse, and 3M (St. Paul, MN), a major FFR manufacturer.

In July 2020, an analysis of existing evidence on decontamination of N95 FFRs using UV-C, ranging from the time when N95s were invented in 1972 to March 2020, concluded that the function of N95 FFRs, based on aerosol penetration and air-flow filtration, was maintained following a single cycle of UV-C exposure [13]. It was also concluded that UV-C could be a successful method of inactivating infectious pathogens on N95 FFRs, provided the effectiveness of UV-C disinfection was validated, and the impact of UV-C on the N95 FFRs’ fit, form, and function for each UV-C use case was determined [13].

By September 2020, extensive literature was available for decontamination procedures for N95 respirators, using either bacterial spore inactivation tests, bacteria, or respiratory viruses (*e.g.*, influenza A virus) (see Fischer *et al.* [14] and references within). Fisher *et al.* [14] analyzed four different decontamination methods, UV radiation (260 nm to 285 nm), 70 °C dry heat, 70% ethanol, and vaporized hydrogen peroxide (VHP), for their ability to reduce SARS-CoV-2 contamination and their effect on N95 respirator function [15]. Their results indicated that, in times of shortage, N95 FFRs can be decontaminated and reused up to three times by using UV radiation and HPV and one to two times by using dry heat, provided decontamination was performed for sufficient time and proper function of the respirators after decontamination was evaluated using readily available qualitative fit testing tools [14]. At that time, the CDC did not have specific recommendations on the minimum UV dose, however a 1 J/cm^2^ dose had been reported to reduce tested viable viral loads by 99.9% (see Fischer *et al.* [15]).

Currently, the CDC does not recommend decontamination and reuse of N95 FFRs so there is not a standard recommended dose, and N95 FFRs should be disposed after each use [9]. However, it is recommended that organizations follow a continuum using the surge capacity approach in the order of conventional (everyday practice), contingency (expected shortages), and crisis (known shortages) capacities. The latter may include decontamination and subsequent reuse of FFRs when FFR shortages exist [9]. For example, Fischer *et al.* [14] reported at times of shortage, N95 FFRs can be decontaminated and reused up to three times by using UV-C radiation. There are currently no manufacturer-authorized methods for FFR decontamination before reuse and only respirator manufacturers can reliably provide guidance on how to decontaminate their specific models of FFRs [9]. In the absence of manufacturer’s recommendations, third parties, such as companies that provide decontamination solutions, safety organizations, or research laboratories, may also provide guidance or procedures on how to decontaminate respirators without impacting their performance [9].

**Fig. 2:**
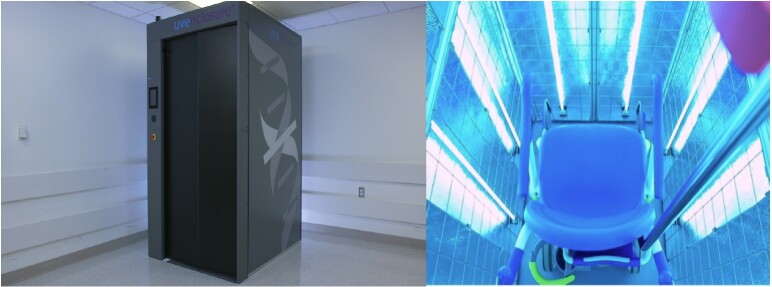
UV-C enclosure (left) and interior (with UV-C lamps irradiating, right). The exterior height, width, and depth of the UV-C enclosure (left) are 88 in (2.2 m), 49 in (1.3 m), and 60 in (1.5 m), respectively. Photo credits: UV-Concepts.

In this paper, we report on an investigation of the use of UV-C for the disinfection of N95 FFRs using an UV-C enclosure (Fig. 2, UV-Concepts, Littleton, CO)^8^8Certain commercial equipment, instruments, or materials are identified in this paper to specify the experimental study adequately. Such identification does not imply recommendation or endorsement by the National Institute of Standards and Technology, nor does it imply that the materials or equipment identified are necessarily the best available for the purpose.. The UV-C enclosure optimizes the key principles of UV-C light disinfection effectiveness for high-touch portable medical equipment. It is currently not marketed for N95 FFR disinfection and reuse.

The evaluation of UV-C for N95 FFR disinfection and reuse using the UV-C enclosure began at the onset of the COVID-19 pandemic in collaboration with NIST in support of its efforts to address the COVID-19 pandemic through the use of PPE.^9^9Other areas NIST provided metrology and expert input to address the COVID-19 pandemic included: biological measurements; machine learning, artificial intelligence, data, and analytics; ventilators, manufacturing and industry, ventilators, wireless innovations, energy and environment, and technology transfer resources. See https://www.nist.gov/coronavirus for more information (accessed February 11, 2022). As the pandemic response grew, heavy burdens were placed on acute- care hospitals through the needs for increased critical care capacity and modified use of PPE [16].

UV-Concepts and ResInnova Laboratories (Rockville, MD) managed the procedures for operating and testing the UV-C enclosure for N95 FFR disinfection and reuse. ResInnova Laboratories performed the operation procedures and viral inactivation tests. NIST conducted physical metrology studies of the UV-C radiation delivery within the UV-C enclosure. NIST also performed N95 FFR material characterizations to assess metrology needs to understand material integrity and performance of N95 FFRs following UV-C irradiation. Material integrity was investigated by NIST using high resolution scanning electron microscopy (SEM) for surface microanalysis inspection of the irradiated N95 FFRs. Tensile strength, gas flow, and particle filtration were also examined by NIST using test methods and equipment developed at NIST. Performance and fit of the irradiated N95 FFRs were also investigated by the National Personal Protective Technology Laboratory (NPPTL) research center within NIOSH (Pittsburgh, PA) using established NPPTL protocols.^10^10U.S. Code Title 29 U.S.C. 651 et seq.; 30 U.S.C. 3, 5, 7, 811, 842(h), 844; U.S. Code of Federal Regulations, Title 42 CFR part 84— Approval of respiratory protective devices (see footnote 3); *Federal Register*, Vol. 60, 30355, June 8, 1995, unless otherwise noted. See https://www.govinfo.gov/app/details/USCODE-2010-title29/USCODE-2010-title29-chap15-sec651 for more information.

Results from the operational and metrological tests are presented in this article. The results are not intended to be used to quantify the safety of masks or the ability of UV radiation to disinfect used masks^11^11It is important to note the evaluations and results in this work are specific to a manufacturer’s UV-C enclosure and a manufacturer’s make and model type of N95 FFRs as described in Sec. 2.1. These results cannot be generalized to other UV irradiation strategies, devices, or chambers or generalized to any N95 FFR. Any disinfection and reuse strategy for N95 FFRs must test and evaluate the specific equipment and N95 FFRs for efficiency, effectiveness, and reuse. but rather these results are intended to provide information on the use of UV-C for disinfection and potential effects of UV-C radiation on the N95 FFR material properties and performance. These results will be useful for the UV-C radiation industry in the development and deployment of UV-C technology in the medical field for N95 FFR disinfection and reuse strategies. This work also supports the needs identified at a NIST workshop on the use of UV technologies for public health that took place in January 2020 [4, 17], which was just prior to the onset of the COVID-19 pandemic.

Since the NIST workshop in January 2020, the metrology and best practices for deploying UV technologies for disinfection in many applications are reported in a special section of the *Journal of Research of the National Institute of Standards and Technology* on UV technologies for public health [4], including PPE [18–20], whole rooms [21], aircraft [22], surfaces [23–25], and air [26, 27]. Metrology was also reported for understanding UV photonics [28], reflectivity of UV from materials [29], the delivery and distribution of UV to targets [30], and the UV inactivation of biological targets [6, 31].

## 2 Methods and Results

### 2.1 Irradiation and Virus Inactivation (ResInnova Laboratories)

OC43, a betacoronavirus (ATCC VR-1558), was propagated in rhabdomyosarcoma cells (RD; ATCC CCL-136) to a titre of approximately 1 × 10^8^ viral particles/mL as determined by both focus-forming and plaque-forming assays, as previously described [32]. For focus-forming assays, infected RD cells were dried and fixed with an organic fixative. OC43 nucleocapsid protein-specific mouse monoclonal antibodies (clone 541-8F; Sigma-Aldrich) were added to the fixed cells, and indirect immunostaining of virus-infected areas of cellular monolayer (foci) was determined using a Vectastain ABC kit following the manufacturer’s instructions. Viral foci were enumerated using a hand lens and verified, when needed, using a light microscope. All values were reported as focus-forming units (FFU).

**Fig. 3:**
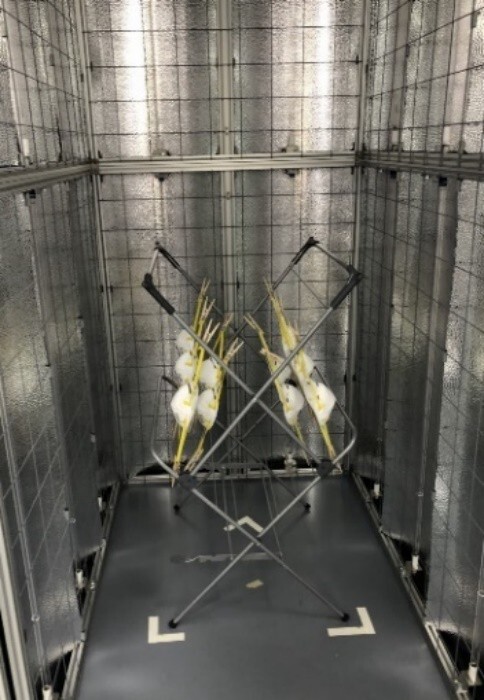
Set-up of the N95 FFRs in the UV-C enclosure for irradiation. The N95 FFRs were clipped to a rack with the exterior surfaces of the masks facing towards the lamps. The masks were also staggered on the rack to provide exposure both on the outside and inside of the masks.

The exterior areas of two N95 FFRs^12^12The N95 FFRs evaluated were new and from commercial vendors labelled as “N95” and manufactured by 3M (model number 1860). Only two N95 FFRs were used for the virus inactivation tests due to the shortage of new, unused N95 FFRs at the time of this evaluation. See footnote 2 and Ref. [2] for information on the N95 designation for FFRs and Fig. 1 for information on how N95 FFRs differ from facemasks (sometimes called surgical masks)). Also see the disclaimer information in footnote 8 regarding the mention of commercial equipment, instruments, or materials. were inoculated with 4.55 × 10^6^ viral particles of OC43 in 10 uL of inoculum buffer at four locations (20 mm circles; locations described in Table 1). The inoculum buffer was composed of Eagle’s modified essential medium (EMEM) supplemented with a 2% fetal bovine serum. The N95 FFRs were then pinned to a drying rack inside the UV-C enclosure (Fig. 2) such that the exterior of the masks faced either side of the enclosure (Fig. 3).

The UV-C enclosure was then operated for one 3 min interval to achieve a dose of approximately 1000 mJ/cm^2^. The CDC reported that a 1 J/cm^2^ dose can reduce tested viable viral loads by 99.9% [15].

The irradiance of the UV-C enclosure was measured in the physical center of the chamber using an International Light Technologies ILT2400 system with a narrow band 254 nm sensor. The calibration of the sensor was confirmed as described in Sec. 2.2. UV-C radiation was generated at 254 nm from low-pressure mercury that were contained within the UV-C enclosure. There were 19 lamps positioned within this enclosure: six lamps on each side wall, four on the rear wall, and three on the ceiling. The interior side of the door had a highly reflective surface, and each lamp was surrounded by a curved highly reflective material. This enclosure design minimizes shadowing, which allows for 360°irradiation exposure to the target subject (see Fig. 3).

Ten 3 min intervals were additionally conducted as described above for non-viral laden N95 FFRs which were reserved for physical metrology evaluations by NIST and NPPTL (see Secs. 2.3–2.7). A set of N95 FFRs was also put aside as non-irradiated controls. The purpose of repeating the intervals was to extensively expose the materials comprising the N95 FFRs to UV radiation and compare these with the controls. Sets of the non-viral laden irradiated N95 FFRs and non-irradiated N95 FFRs were placed in commercial plastic bags, packaged in a box, and then transferred to NIST for materials and performance metrology investigations as described in Secs. 2.3–2.7. NIST subsequently transferred a set of seven non-viral-laden irradiated N95 FFRs and three non-irradiated N95 FFRs to the NPPTL (see Sec. 2.7).

**Table 1:** UV-C inactivation of OC43.

Spot Location	Sample Name	Average Virus Recovered (FFU)	Average Log Recovery	Average Log Reduction (*vs.* Control)	Average % Reduction (*vs.* Control)
Control	Layer 1	9.00E+04	4.95	Not applicable (n/a)	(n/a)
	Layer 2	5.00E+02	2.70	(n/a)	(n/a)
	Layer 3	1.75E+02	2.24	(n/a)	(n/a)
Spot 1—Top of mask, closest to nose bridge	Layer 1	7.50E+01	1.88	3.07	99.92
	Layer 2	7.50E+01	1.88	0.82	85.00
	Layer 3	7.50E+01	1.88	0.36	57.14
Spot 2—Top middle of mask	Layer 1	7.50E+01	1.88	3.07	99.92
	Layer 2	0.00E+00	(n/a)^a^	2.70^b^	100.00
	Layer 3	0.00E+00	(n/a)	2.24	100.00
Spot 3— Bottom middle of mask	Layer 1	0.00E+00	(n/a)	4.95	100.00
	Layer 2	0.00E+00	(n/a)	2.70	100.00
	Layer 3	0.00E+00	(n/a)	2.24	100.00
Spot 4— Bottom of mask, closest to chin	Layer 1	1.50E+02	2.18	2.77	99.83
	Layer 2	0.00E+00	(n/a)	2.70	100.00
	Layer 3	7.50E+01	1.88	0.36	57.14

a *Log 0 is an undefined number

b Because log 0 is an undefined number we have substituted the real number 0 for the log of 0 in this calculation.

Post-exposure, for the viral-laden N95 FFRs, the inoculated areas were cut out of the mask and separated into their constituent three layers. Each layer was recovered into soya casein digest lecithin polysorbate (SCDLP) buffer by vortexing. Dilutions of the recovery solution were plated onto RD cells and incubated at 37 °C in 5% CO_2_ for 1.5 h. The liquid was aspirated, and a mixture of 1% agarose with 2% fetal bovine serum and complete EMEM was overlaid onto the cells. Cells were then incubated at 37 °C in 5% CO_2_ for 24 h. The agarose was removed, and the cells were dried and fixed prior immunostaining as stated above. The resulting immuno-foci were enumerated as FFUs, and log reduction values relative to an unexposed control mask were calculated.

Post-inoculation examinations revealed that the OC43 was distributed through all three N95 FFR layers although it was largely contained within the outer layer (Table 1). See Sec. 2.3 for more information on the N95 FFR layers, including a photograph and high-resolution SEM images.

Following UV-C irradiation of the viral-laden N95 FFRs, the outer layers all had greater than 99% reduction of virus activity. The N95 FFR topography had a clear impact on UV-C irradiation and viral inactivation. The area nearest the chin, which has a steep surface angle relative to the UV-C source, had the lowest inactivation of 99.83%. The other areas had 99.92% to 100% virus inactivation. The UV-C penetrated to both the second and third layers of the N95 FFRs, based on the surrogate of viral recovery. In the second layer, UV-C irradiation inactivated between 85% and 100% of OC43. Virus inactivation was lower in the third layers (as low as 57.14%) (see Table 1). Based on the FFU data, it was concluded that UV- C irradiation was effective at inactivating OC43, even through multiple layers of the N95 mask with up to 100% reduction.

### 2.2 Dosage and Sensor Calibrations (NIST)

The irradiance in the UV-C enclosure was measured in the physical center of the chamber using an International Light Technologies (ILT) ILT2400 system with a narrow band 254 nm sensor (XSD140T254). The accredited calibration report provided by ILT states that the expanded uncertainty for optical radiation responsivity is 6.9% (*k*=2). The calibration is traceable to the International System of Units (SI) through NIST. The ILT calibration was confirmed by NIST through a direct comparison to a stray-light-corrected spectroradiometer calibrated against deuterium lamps calibrated by NIST. Both detectors were illuminated by a low-pressure Hg lamp.

The spectroradiometer uses a cooled, back-thinned charge-coupled device (CCD) with spectral range from 200 nm to 800 nm and bandpass of 2.5 nm. The irradiance probe is a 12.7 mm diameter UV diffuser that has a near-cosine angular response. A 3 m optical fiber bundle is used to couple the incident light from the irradiance probe to the spectroradiometer. A tube baffle is placed in front of the irradiance probe to limit its field of view to be within the black velvet board to minimize the scattered room light. The reflectance of the black velvet is measured to be less than 1% from UV to near infrared.

The spectroradiometer was calibrated against a transfer standard deuterium lamp from 200 nm to 400 nm for spectral irradiance responsivity [33]. The signal-level nonlinearity of the spectroradiometer was characterized and corrected. In addition, the spectroradiometer was also characterized and corrected for stray light using the matrix method [34]. The correction for stray light is critical because stray-light errors are significant, and the spectrum of the low-pressure Hg lamp and that of the deuterium lamp are very different.

The UV responsivity of the spectroradiometer had an expanded uncertainty of 2% (*k* = 2). The ILT device measured 2% lower than the calibrated spectroradiometer, which is well within the uncertainty of the comparison.

A simplified model of the UV-Concepts UV-C enclosure was developed using the OpticStudio optical design and illumination analysis program [35]. The model allows for an estimation of the fluence rate [36] anywhere within the enclosure, given assumptions for the internal wall reflectance and the consistent emitted power of each lamp [37]. The fluence rate, also known as spherical irradiance, is the mean value of irradiance on the outer curved surface of a very small (real or imaginary) sphere at a point in space. Therefore, the virtual detectors collect over a 4 π solid angle.

**Fig. 4:**
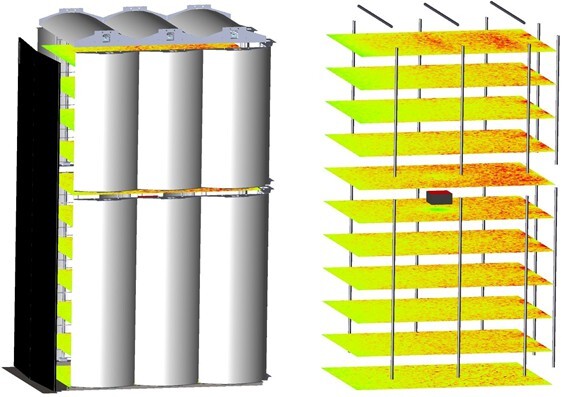
UV-C chamber model (left) and virtual detectors reporting fluence rate (right).

Referring to Fig. 4, at left, the interior of the chamber including 19 lamp reflectors is shown. The chamber door is shown in black. At right, the 19 lamps are clearly seen, and 11 virtual detector planes are arranged in a shelf-like pattern at various heights within the enclosure. For clarity, the lamp reflectors are not shown. Each detector reports fluence rate in false color. In the center, a small box is located at the center of the middle “shelf.” At its top in red, another virtual detector is shown, representing the real detector used in these experiments. The virtual detector representing the real detector reports irradiance [38]. Irradiance is the density of incident radiant flux with respect to area at a point on a real or imaginary surface. In this case, the area was a flat surface.

**Fig. 5:**
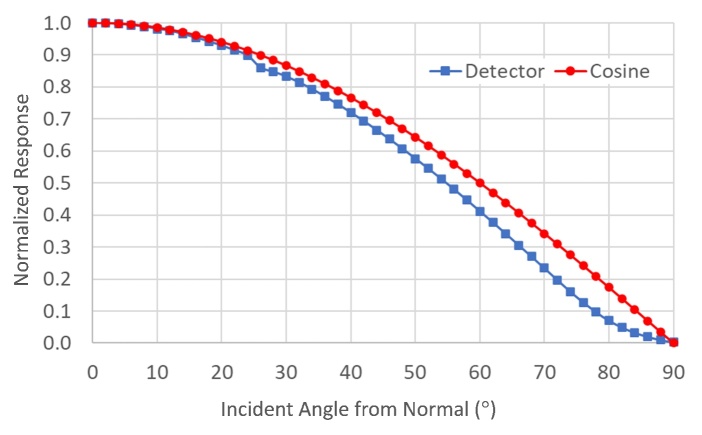
Angular response of the detector (blue square) compared to an ideal cosine response (red circle).

Using this simulation, the fluence rate can be estimated anywhere in the chamber based on the irradiance measured with the real detector. Typically, in a simulation of this nature, an assumption is made that the angular responsivity of the detector is ideal, that is, a true “cosine-corrected” response. In the visible region of the spectrum, a variety of materials can accomplish high-quality cosine corrections. In the UV-C region, the number of materials is more limited, and the cost of this optical component can be a large aspect of the final price of a detector. The angular response was measured for this detector using four orthogonal axes and is presented in Fig. 5.

**Fig. 6:**
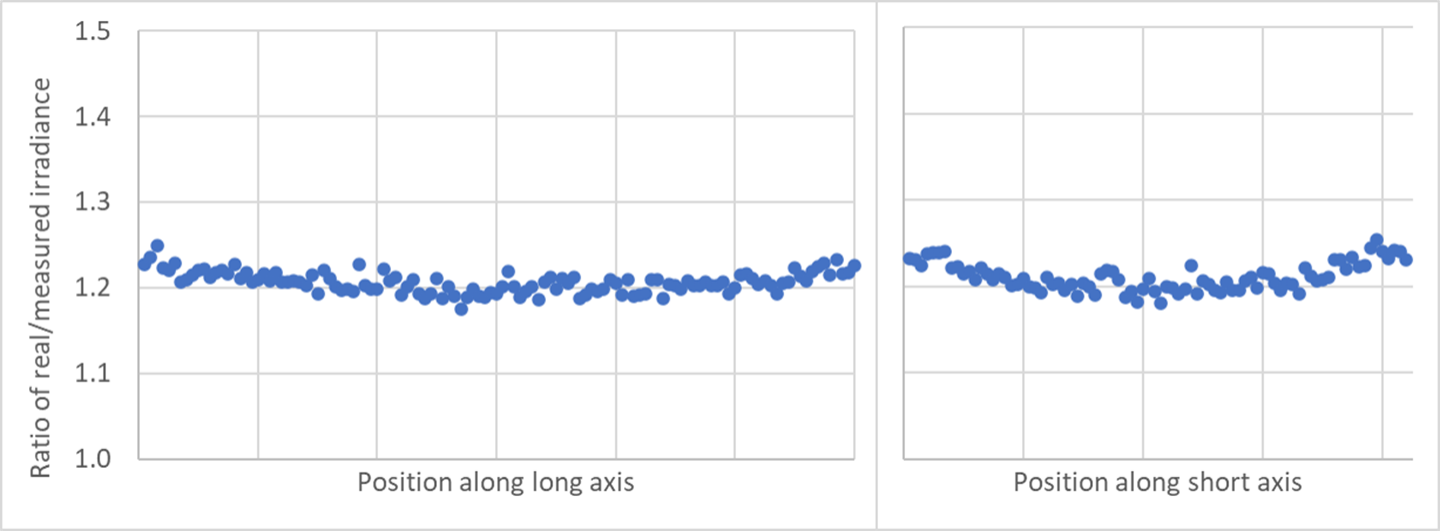
Ratio of real irradiance divided by the measured irradiance of the real detector due to the angular response of the real detector.

Figure 6 shows the simulated results for the ratio of the real irradiance divided by the as-measured irradiance, with the angular response of the detector incorporated, plotted over the entire plane of the middle shelf. The granularity is due to the finite number of rays traced and could be smoothed if more rays, with a corresponding increase in computation time, were used. The simulation is the result of 15 million random rays from each lamp in the chamber. Since the larger angles from normal do not provide the expected signal, the signal measured by the detector was multiplied by 1.20 to compensate.

**Fig. 7:**
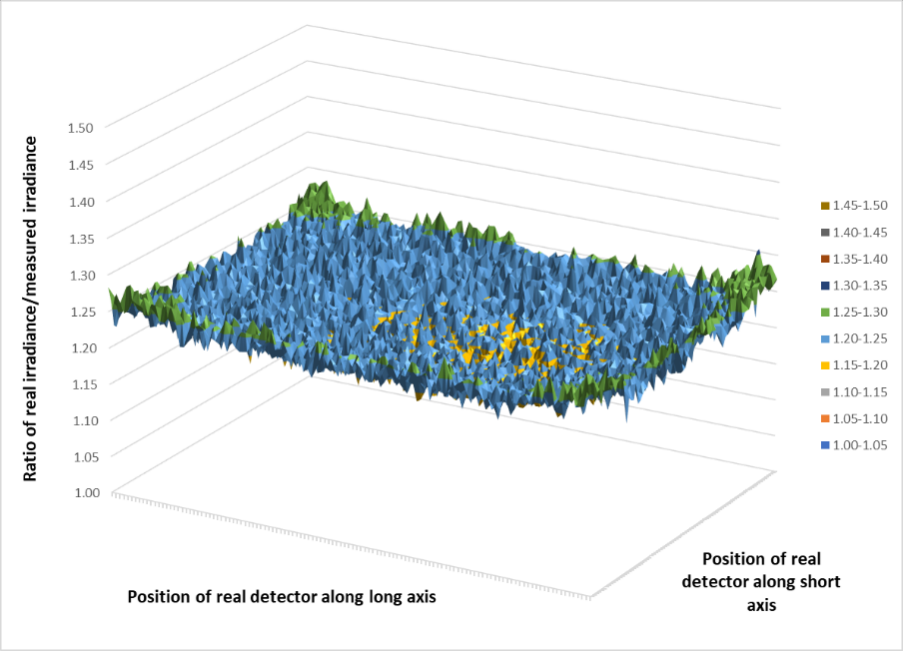
Slice of real/measured irradiance at the middle shelf.

Figure 7 shows a slice through the center of the short axis (left) and a slice through the center of the long axis (right). The standard deviation for the simulation ratio is 0.01; however, the uncertainty of the ratio due to potential imperfections in the model places the estimated expanded uncertainty at 10% (*k* = 2) for the ratio. Including the calibration of the detector, the expanded uncertainty for the fluence rate within the chamber is 12% (*k* = 2).

### 2.3 High-Resolution Scanning Electron Microscopy (SEM) Measurements (NIST)

**Fig. 8:**
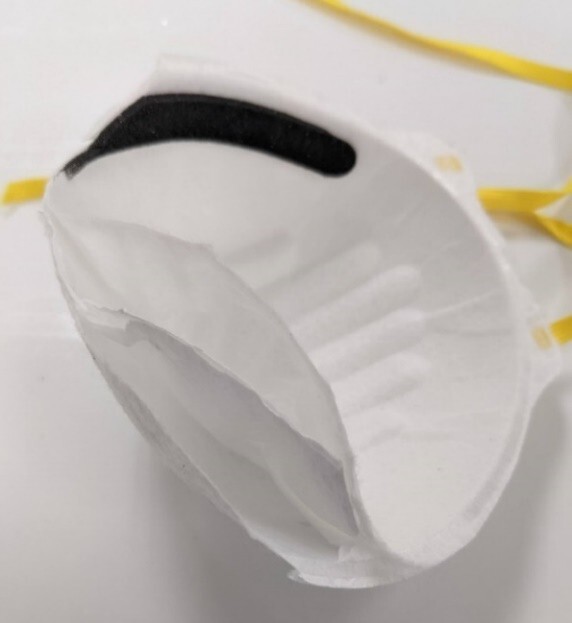
Photograph of the three layers comprising a N95 FFR. Photo credit: Used with permission from 4 C Air.^14^14Zhao M, Poster D, 4 C Air (2022) Personal communication.

High-resolution SEM was used to assess the surface structure of the various filtering filaments within the N95 FFRs before and after UV-C irradiation. A description of how the N95 FFRs were irradiated is in Sec. 2.1. To make SEM imaging of the insulating material comprising the filaments possible, a 7 nm to 10 nm thin contiguous osmium (Os) layer was deposited using a suitable Os deposition system [39]. Images at about 60× magnification to over 2000× magnification were acquired on the N95 FFR filaments visible on the surfaces on the three layers comprising the N95 FFR (Fig. 8).^13^13N95 FFRs typically have three layers consisting of a polypropylene/polyester filter with a polypropylene shell and polypropylene cover web in the shape of a rigid cup with at least two elastic loop bands attached [40]. See the technical specifications for more information regarding the 3M 1860 N95 FFRs at https://multimedia.3m.com/mws/media/1425065O/tech-spec-3m-healthcare-particulate-respirator-and-surgical-mask-1860-n95.pdf. Some SEM images were also taken after the N95 FFRs were subjected to flow resistance testing (see Sec. 2.4) to investigate if the surfaces of the various layers changed after the flow resistance testing was conducted.

High-resolution SEM sample preparation consisted of cutting out approximately 7 mm by 15 mm portions of the N95 FFRs with a sharp blade at their top, separating the three (outer and inner particle filters and the center, electrostatic fine particle filter) layers, positioning them on a flat 75 mm diameter Al sample holder and securing them with phosphor bronze springs at their two ends. All layers were prepared this way for their outer and inner surfaces; *i.e.,* altogether, six strips were prepared from each N95 FFR. Several non-used N95 FFRs were examined. Some were irradiated in the UV-C enclosure and others were not. Also, one N95 FFR that had been used for 10 h was examined to investigate if the surfaces of the N95 FFR changed with use. None of the N95 FFRs examined by SEM contained intentionally deposited viruses.

After Os deposition, the prepared samples were placed into the SEM [41] for secondary electron (SE) imaging. For the acquisition of SE images, the following parameters were used: 5 keV landing energy; 43 pA primary electron beam current; in-lens SE detector with no protruding magnetic field column setting. SE images were taken at 1024 (horizontal) by 884 (vertical) pixel resolution, and several images were taken at resolution four times that to allow for digital magnification of these images to explore details later. These 8 bit images were acquired so that the histograms spanned over at least 220 gray levels, with no or only a few saturated pixels. The images were saved in Tagged Image File Format (TIFF) files to avoid distortions caused by glossy image compression.

**Fig 9:**
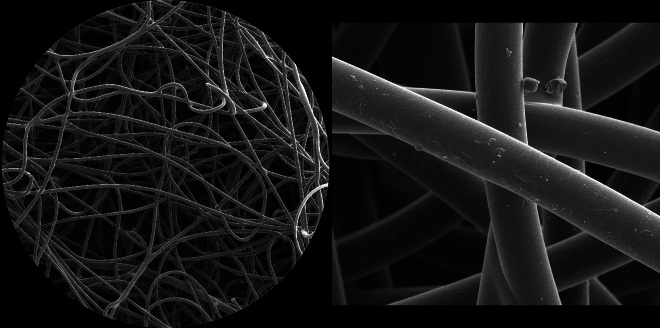
SE images of the surface of the outer layer (*i.e.*, the layer furthest away from the user’s face) of an unused, non-irradiated N95 mask; 2.2 mm HFW (left) and 127 μm HFW (right). The fibers are approximately 18 μm in diameter.

High-resolution SEM results showed no detectable changes in the N95 FFR filaments due to UV-C irradiation. Figure 9 shows SE images of the outer surface of an unused, nonirradiated N95 FFR, with a 2.2 mm horizontal field width (HFW) SE image on the left and a 127 μm HFW SE image on the right. These HFW values correspond to 60× and 1000× magnification on a 4 inch (101.6 mm) by 3 inch (76.2 mm) size photo. The filaments’ diameters look similar, but Y-shaped forks are visible. The surfaces of the filaments show small-size particles and even smaller depositions and some surface unevenness or roughness. The uneven pore sizes vary from about 300 μm to about 10 μm. The “maze” formed by these filaments slows down the air flow and effectively filters particles down to sizes of about a couple of micrometers.

**Fig. 10:**
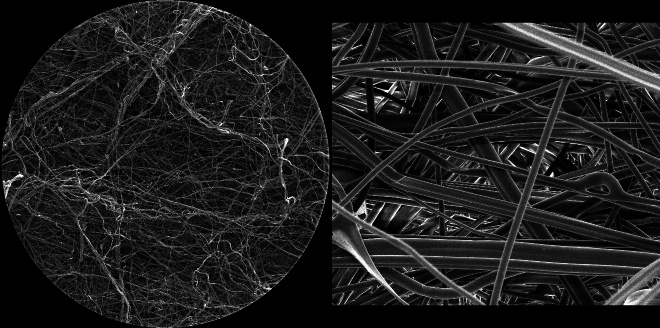
SE images of the center, electrostatic layer filter surface of an unused, nonirradiated N95 mask: 2.2 mm HFW (left) and 127 μm HFW (right). The diameters of individual filaments vary from about 1.4 μm to about 5.4 μm.

Figure 10 shows 2.2 mm HFW (left) and 127 μm HFW (right) SE images of the center, electrostatic layer filter surface of an unused, non-irradiated N95 FFR. These filtering filaments are much smaller in diameter and form a much denser filter relative to the outer filter at the front of the N95 FFR (see Fig. 9).

The center filter can capture with high efficiency small water droplets that potentially carry pathogens, such as a virus. The size and shape of the filaments vary more and show larger surface areas than simple cylindrical cross-section filaments. This denser pattern provides a filter that slows down air flow, and the larger, electro-statically charged surfaces forming the pattern provide the advantageous capability for filtering sub-micrometer-sized water droplets and particles.

**Fig. 11:**
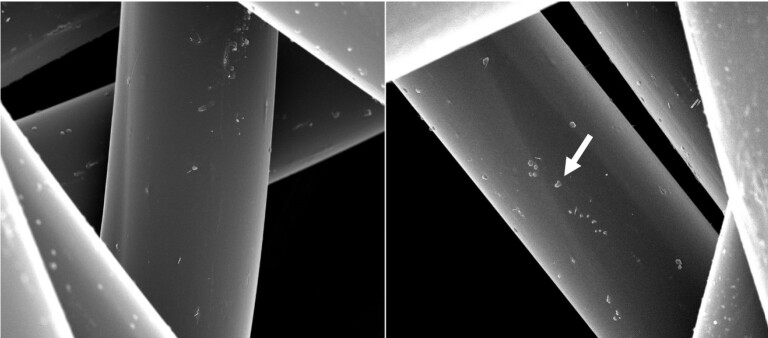
SE images of the surfaces of the inner front layer (closest to the user’s face) of unirradiated (left) and irradiated (right) N95 FFRs, with 50.8 μm HFW for both images. There is no visible difference. The arrow points at an approximately 200 nm size particle.

Figure 11 shows 50.8 μm HFW SE images of the front surfaces of unirradiated (left) and irradiated (right) masks. There is no visible evidence that would make it possible to differentiate the two.

**Fig. 12:**
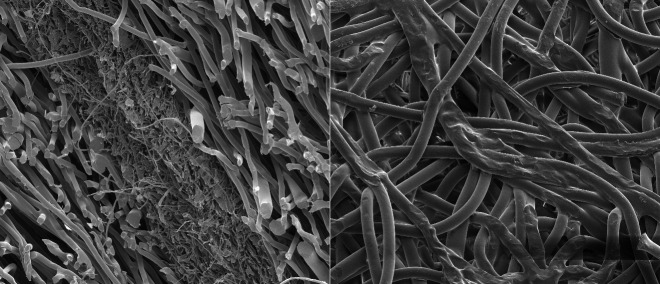
SE images of the cross section (left) and heat-treated, fused rim (right) of an unirradiated N95 FFR, with 1.06 mm and 508 mm HFW, respectively.

Figure 12 provides an SE image of the cross section of an unirradiated N95 FFR to show the density and fiber size differences of the three layers. The image on the right was acquired from the heat-treated rim of the N95 FFR, showing the heat-fused filaments in that region, which provide the shape and secure fit of the N95 FFR.

**Fig. 13:**
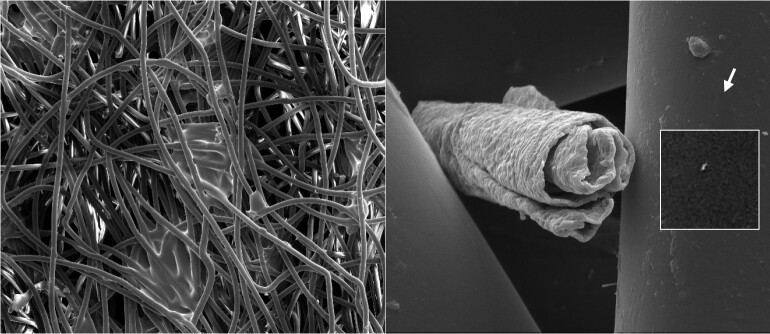
SE images of an N95 mask after 10 h of use by a human (this was not an irradiated or viral-laden N95 FFR). Left: Inner, mouth side, showing some deposited, conceivably mucus-type material, with 1.27 mm HFW. Right: Front side, showing the fine surface variations of the filter fibers and an unknown, possibly plant-origin particle, with 25.4 μm HFW. These images demonstrate the extent to which the NIST SEM capabilities can discern images of contaminants in the layered structure of the N95 FFRs.

Figure 13 shows images from the unirradiated N95 FFR that had been used for 10 h in a clean laboratory environment where there was no significant source of dust/airborne contaminants. The left image was taken on the inner front layer (closet to the user’s face and mouth). Here, some deposited, conceivably mucus-type material is visible. The image on the right was taken on the outer layer of the mask. The fine surface variations of the filter fibers are clearly visible. Due to the high cleanliness of the laboratory air where the SEM was operated, no noticeable number of extra particles was observed compared to never-used clean masks. Still, an unknown, possibly plant-origin particle was found. The arrow points at an approximately 80 nm by 100 nm size particle, which is also shown in the insert in a digitally magnified version. For comparison, the SARS-CoV-2 virus is about 150 nm in diameter [42].

In summary, the SEM images proved to be useful in the assessment of morphological effects of UV-C irradiation on N95 FFRs. No discernible evidence was found to indicate that the UV-C irradiation in the dose suitable for disinfecting the N95 FFR masks in the UV-C enclosure would damage or alter the fibers of any of the three layers comprising the N95 FFR. The SEM images of this study are available in their original digital form.^15^15Contact author: Andras Vladar (andras.vladar@nist.gov)

### 2.4 Flow Resistance Measurements (NIST)

**Fig. 14:**
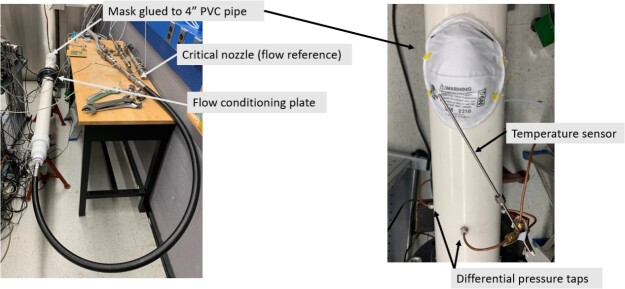
Test set up used for flow resistance measurements at NIST. 4 inches = ~ 10 cm.

The NIST Fluid Metrology Group tested whole N95 FFRs for flow resistance by establishing a known flow and measuring the differential pressure across N95 FFR. The test set up used to measure flow resistance is shown in Fig. 14. Compressed, dry air flowed through a 1.6 mm critical flow venturi (CFV) to provide a reference flow with expanded (*k* = 2, approximately 95% confidence level) uncertainty of 0.1% [43]. The output of the CFV flowed through a 25 mm black flexible pipe to a 100 mm white polyvinyl chloride (PVC) pipe with the end capped. An oval-shaped hole slightly smaller than the mask was cut in the side of the 100 mm pipe and each mask was in turn glued over the hole. A perforated plate installed upstream from the mask between two flanges in the PVC pipe broke up the jet produced by the 25 mm to 100 mm pipe enlargement.

The high-pressure side of a differential pressure sensor was connected to a manifold of four wall taps in the PVC pipe and the low-pressure side was open to the room. The pressure drop with no mask installed was negligible (< 0.06 Pa) and the expanded uncertainty of the differential pressure measurement across the mask was 0.25% of the reading. The temperature and absolute pressure of the gas exiting the mask were used to calculate the gas density and convert the mass flow measured by the CFV to the actual volumetric flow through the mask. The gas temperature was measured by a thermistor placed approximately 25 mm away from the external surface of the mask. The mask was glued over the hole in the PVC pipe using a hot glue gun. The glue joint was tested for leaks by pressing the edge of the mask down by hand and observing whether the differential pressure increased. If it did, the glue joint was reinforced with more glue.

**Fig. 15:**
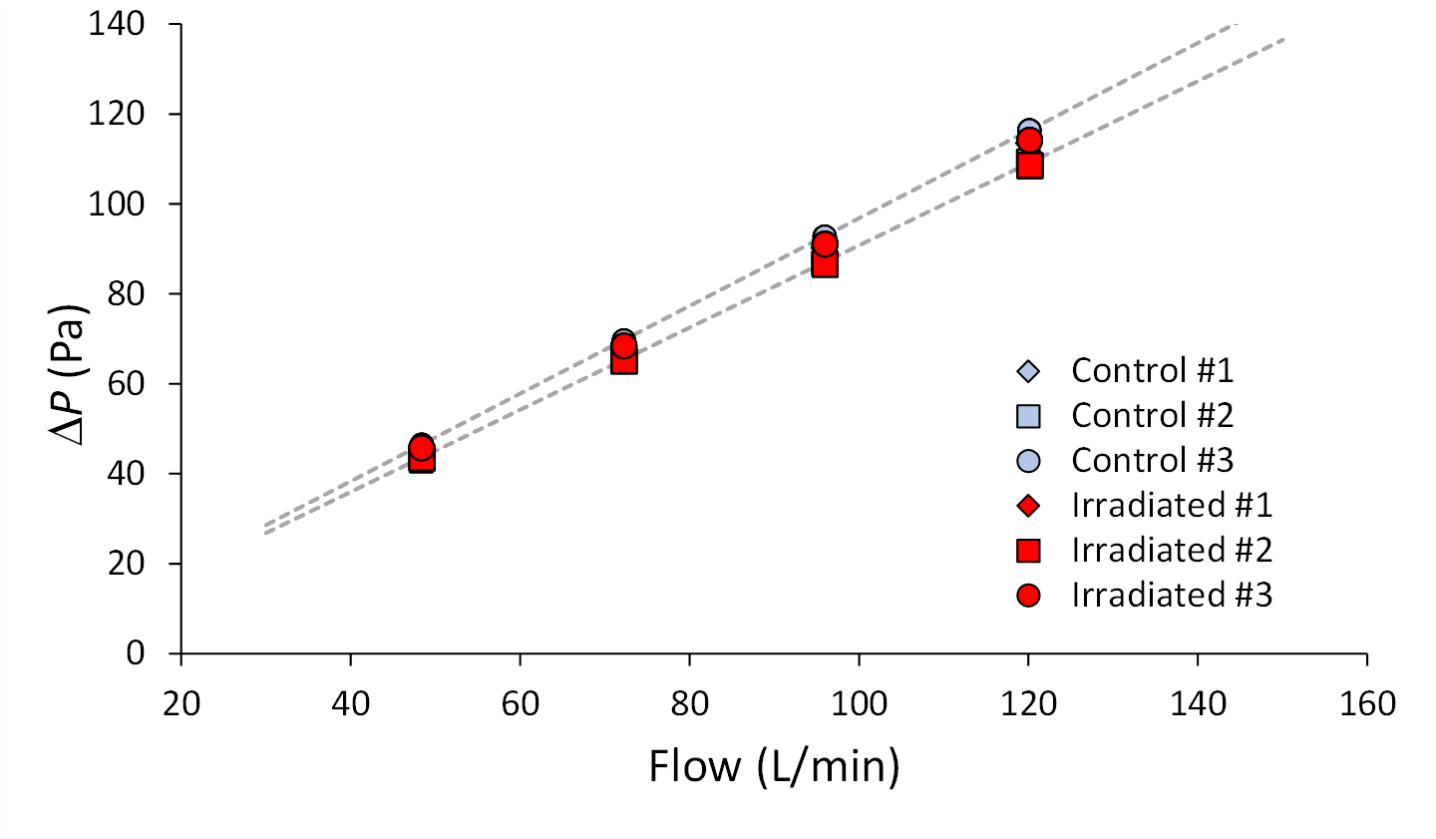
Pressure drop (Δ*P* (Pa)) *vs*. flow (L/min) for three irradiated and three control masks and example linear best fits (dashed lines) to the experimental data from two of the six masks. The pressure drops for flow of 85 L/min were used to assess whether the masks were changed by the UV-C irradiation.

Six N95 FFR masks were tested, three irradiated in the UV-C enclosure and three controls see Sec. 2.1). The pressure drop was measured at five flows between 48 L/min and 120 L/min. At each flow set point, five 30 s long averages of the CFV flow, pressures, and temperatures were gathered. The five points were averaged to produce the values plotted in Fig. 15. Linear best fit equations were used to interpolate the pressure drop for the flow specified in the mask performance requirement (85 L/min).

**Fig. 16:**
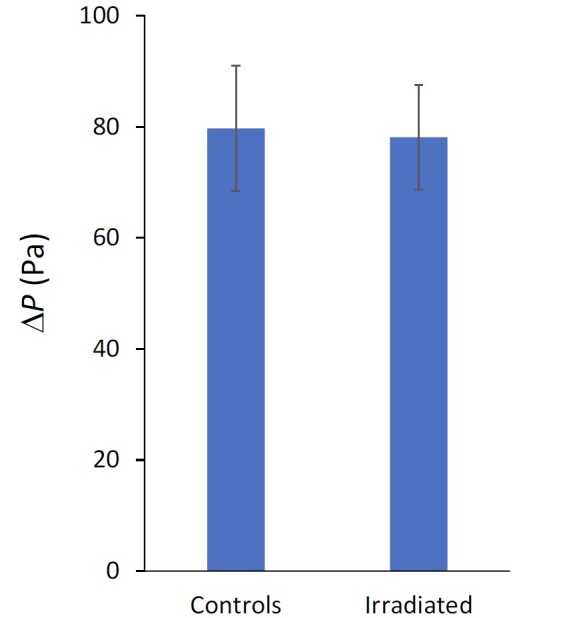
Pressure drop (Δ*P* (Pa)) for N95 FFRs at 85 L/min. See the text for a description of the uncertainties that are represented by the error bars.

The average pressure drop for the two sets of three masks plotted in Fig. 16 showed no significant change in the flow resistance due to the UV-C treatment. The 95% confidence level uncertainty bars in Fig.16 include type B uncertainties due to the flow, pressure, and temperature measurements as well as type A, statistically determined uncertainty. The type A uncertainty accounts for > 99% of the uncertainty due to the small number of masks available. The *t*-test coverage factor was 4.3 for a sample size of three. No discernible evidence was found that the UV-C irradiation in the dose suitable for disinfecting the N95 FFR masks in the UV-C enclosure had an effect on the N95 FFR flow resistance or increased the difficulty for a wearer to breathe through the masks.

In preliminary tests, 30 coupons, 2 cm in diameter each, were cut out of a single, un-irradiated N95 FFR and their flow resistances were measured. We abandoned the coupon approach and decided to test whole masks instead because of the large variance in the resistance of coupons cut from the same mask. The standard deviation of the coupon resistance values was 13%, and the resistance was correlated with the thickness of the mask material, which varies due to the mask manufacturing process.

### 2.5 Tensile Strength Measurements (NIST)

Changes in the mechanical properties of the materials used in the N95 FFRs have the potential to lead to degraded performance of the respirators. While complete tearing of the material while in use may be unlikely, a reduction in the strength or stiffness (corresponding to a decrease in the tensile modulus) has the potential to allow the respirator to deform more than intended, allowing increased leakage between the edges of the respirator and the wearer’s face and thereby compromising the protection that the respirator provides. If the strength of the material were to decrease too much, there is a risk of the material tearing during donning or doffing. Such a failure is not likely to put the wearer at risk, but when there are extreme shortages of PPE, such as during the early phases of the SARS-CoV-2 pandemic, replacements may not be available.

Tensile testing of material samples taken from N95 FFRs was used to determine if the exposure to UV-C radiation would significantly change the strength of the N95 FFR materials. The tensile tests were performed using a universal test machine (UTM) operating in constant-rate-of-extension mode. This test machine consists of an Instron 5800 series electro-mechanical load frame with an MTS ReNew control system. Tests were be performed according to ASTM D5035-11 (2019), “Standard Test Method for Breaking Force and Elongation of Textile Fabrics (Strip Method)”^16^, with some minor variations to accommodate samples taken from finished products, rather than samples taken from a roll of fabric. Additional N95 FFR fit and strap tensile strength measurements were conducted by the NPPTL (see Sec. 2.7).

One variation from the standard test method was the use of a video extensometer to measure the strain in each specimen during the test. An MTS Systems Corporation (Eden Prairie, MN) Advantage Video Extensometer (AVX) tracked the positions of three pairs of fiducial marks during each test, which provided an estimated strain that corresponded to the applied tensile force.

Tensile test specimens were prepared by cutting sections from the sample respirators, separating the individual layers, and then cutting strips from each layer. The respirators are constructed with a horizontal seam running the full width of the respirator at approximately the mid-point. This seam represented a discontinuity that would likely have influenced test results, so it was decided that specimens should not include the seam. This limited the available material to sample to horizontal strips above and below the seam. The sampling process thus consisted of removing the staples that held the elastic headbands, cutting the respirator into two pieces approximately along the horizontal seam, laying the resulting pieces flat, and cutting way extraneous material, including the seamed edges and foam padding at the nose.

These tensile tests are one of many possible mechanical tests that could be used to evaluate changes in the properties of the materials in the N95 FFRs. They consider the strength of the material in a single direction, and at one rate of deformation. In particular, the changes in the response of the materials when they are loaded for an extended period, such as being under stress while being worn for many hours, were not evaluated. Individual layers were tested independently, so changes in the adhesion between layers and the influence of such changes were not considered. The limited number of respirators available for testing constrained how much material could be tested and how many repetitions could be performed.

**Fig. 17:**
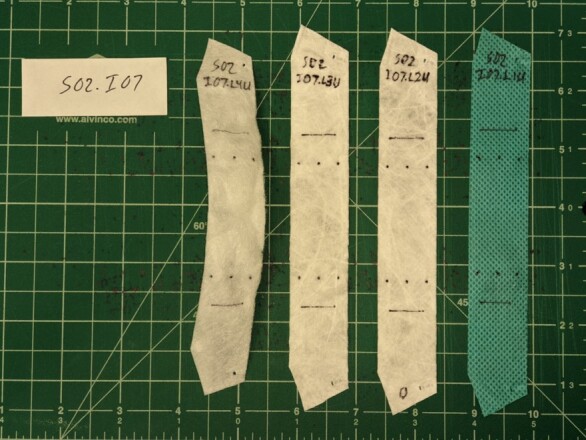
Set of four specimens from an N95 FFR prepared for tensile strength testing. From left to right, the innermost layer, two middle filter layers, and outermost layer.

The sample respirators were constructed with layers of three different materials. The outermost layer was a thin, flexible material. The two middle layers were made from a thin, non-woven material with a relatively low strength. The innermost layer was made from a thicker, stiffer material that appeared to provide most of the support necessary for the respirators to maintain their shape. The technical specifications for the 3M 1860 describe the filter and cover web as polypropylene and the shell as polyester (see footnote 13). Once the extra material was cut away, these four layers in resulting fabric pieces were separated and labeled. Each was then cut into a single 25 mm ± 1 mm wide strip. The specimen lengths varied due to the shape of the respirators, but all specimens had a center section with a consistent width that was at least 125 mm long. Each specimen was marked with fiducial marks to allow strains to be measured with the video extensometer. Three pairs of marks were used, with the upper and lower marks in each pair separated by 50 mm. A typical set of specimens prepared for testing is shown in Fig. 17.

During testing, each specimen was mounted in the test machine and a preload of 0.12 N was applied. The video extensometer was then adjusted to track the six fiducial marks. Finally, the specimen was loaded to failure by applying deformation at a constant rate of 45 mm/min. This rate represents a deviation from the value specified in ASTM D5035-11; however, this adjustment allowed most of the tests to be completed in approximately 20 s and was sufficiently slow to account for the limited data acquisition rate of the video extensometer. The applied force, cross-head extension, and strain were recorded continuously during the test.

The tensile test results indicated that there was no significant change in the material strength due to the UV-C dose that the respirators received. There was relatively large specimen to specimen variability, which was not unexpected due to the nature of fabric samples and the fact that the specimens were extracted from finished products. Each of the three materials had distinct responses, and, therefore, the results from each type of material were analyzed separately. When the results from each set of materials were compared, the changes in the median performance between the irradiated specimens and the control specimens were quite small compared to the variability within each set of specimens.

**Fig. 18:**
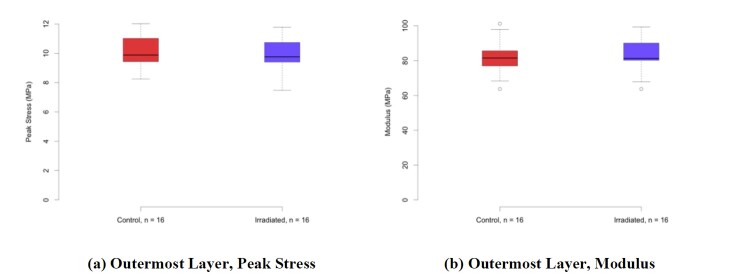

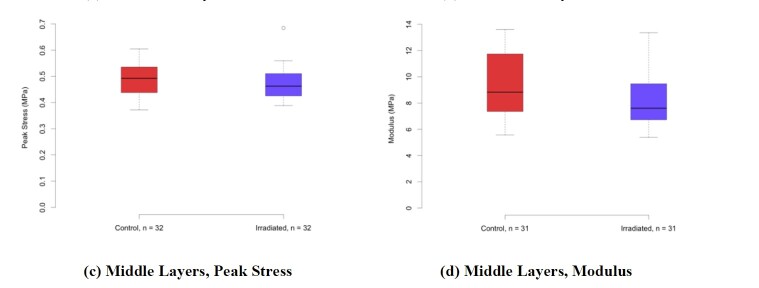

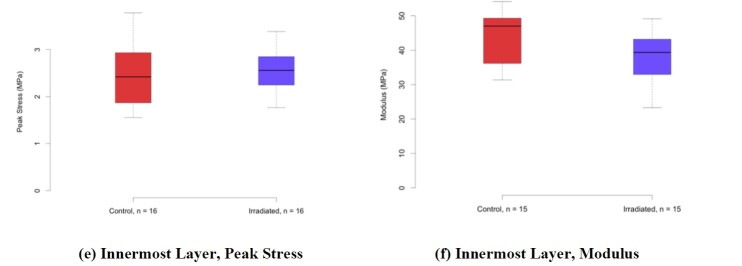
Tensile strength measurement results showing the responses of the specimens from the outer, middle, and inner layers of samples from control (non-irradiated) and irradiated N95 FFRs. The error bars in the box plots represent 1.5× the interquartile range (IQR), or the most extreme data point when there are no data points beyond 1.5× IQR. When there are outliers beyond 1.5× IQR, each one of the outliers is plotted.

Some of the results are shown in Fig. 18. The left column charts show comparisons of the peak stresses, which are the largest stresses observed in the specimens. The specimens tended not to break at the peak stress, but they continued to carry reduced load as the strain increased, and they broke at significantly larger strains. In each chart, the left (red) box in the plot describes the response of the control specimens, while the right (blue) box in the plot describes the response of the irradiated specimens. From top to bottom, the plots show the responses of the specimens from the outer, middle, and inner layers of the respirators. In each case, the median peak stress only changed slightly, and the shift was well within the quartile one to quartile three range.

The right column of Fig. 18 shows comparisons of the fabric modulus in the linear response range. Again, in each chart, the left (red) box in the plot describes the response of the control specimens, while the right (blue) box in the plot describes the response of the irradiated specimens. The median values of the modulus had greater changes than those observed for the peak stress, but the size of the variation was still smaller than the variation within the sample set, and the shifts were not statistically significant.

In summary, from the tensile strength measurement results, it can be reasonably concluded that for the tested respirators the UV-C dose used did not significantly impact the material’s mechanical properties. These conclusions are consistent with the separate tensile strength measurement results reported by the NPPTL (see Sec. 2.7).

### 2.6 Particle Filtration Measurements (NIST)

**Fig. 19:**
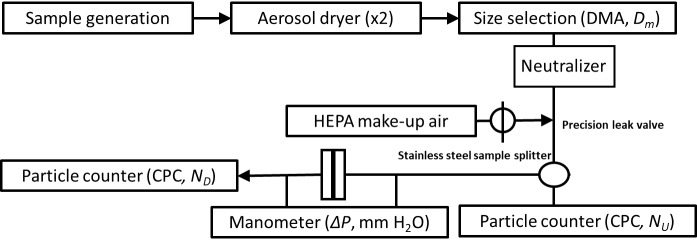
Block diagram for the filtration efficiency experiments. Connections between flows are shown for measurement. Abbreviations: condensation particle counter (CPC) and differential mobility analyzer (DMA). *N*_U_ is upstream particle concentration, and *N*_D_ is downstream particle concentration.

Filtration efficiency (FE) measurements mimicked the mask filtration protocol of the International Organizations for Standardization ISO 29463)^17^17ISO 29463-1:2017 “High efficiency filters and filter media for removing particles from air — Part 1: Classification, performance, testing and marking”. Available at www.iso.org testing standard (where aerosol is reneutralized after size selection) (see Fig. 19). Aerosol was generated from a 2 mg mL^−1^ aqueous solution of NaCl using a constant- output atomizer supplied with dry (dew point < −75 °C), filtered air using a high-efficiency particulate air (HEPA) filter (25 psig (172375 Pa)). Of the 2.2 L min^−1^ flow that was generated, 0.3 L min^−1^ was sampled and conditioned using two silica gel diffusion dryers (desiccant was replaced daily prior to data collection). The aerosol was then passed through a soft X-ray charge neutralizer and size selected, at a mobility diameter (*D*_m_), using a differential mobility analyzer (DMA) and a 10:1 sheath:aerosol flow. Under these experimental conditions, the range of aerosols selected was 50 nm ≤ *D*_m_ ≤ 825 nm.^18^18This range covered the 300 nm size typically reported for filtration efficacy and provided the ability to examine how the material performed across a wide range of particle sizes. The ≈ 0.3 L min^−1^ of aerosol exiting the DMA was then reneutralized using a second soft X-ray charge neutralizer, mixed with ≈ 2.7 L min^−1^ desiccated HEPA-filtered dilution air, and either passed to a condensation particle counter (CPC) or through a 25 mm plastic filter holder (with stainless-steel filter backing) and to a CPC to measure the upstream (*N*_U_) and downstream (*N*_D_) particle number densities, respectively. The relative humidity (RH) and temperature of the airstream flowing through the filter were 20% ± 10% and 23 °C ± 2 °C, respectively. Both CPCs sampled at .5 L min^−1^ and the face velocity at the filter holder was ≈ 6.3 cm s^−1^, in line with NIOSH guidelines. Conductive (carbon black impregnated) silicone tubing was used throughout the experiment to prevent aerosol scavenging. The material under test was held in a plastic filter holder with a polymer gasket that electrically isolated the material. No attempts were made to electrically ground the tested material.

The *FE* was measured and reported as a percentage using the following equation

$FE=100\times \left(1-\frac{N_{D}}{N_{U}}\right)$

by measuring the number density of particles per volume of air (particles cm^−3^) upstream and downstream (*N*_U_ and *N*_D_, respectively) of the filtering material where particles *N*_D_ passed through the filter and particles *N*_U_ were incident on the filter. Data were collected for particles with mobility diameters (*D*_m_) between 50 nm and 825 nm, which is consistent with the method described in ISO 29463.

The downstream CPC had been previously calibrated by a spectroscopic method using ammonium sulfate aerosol [44]. Using this method, the CPC calibration was better than 3% for number densities (*N*) < 2× 10^4^ cm^−3^. At larger values of *N*, *N*_U_ and *N*_D_ deviated from each other, so they were compared multiple times daily using the described arrangement without fabric in the filter assembly. Both CPCs (TSI 3775) also switched between a single particle counting mode and a photometric counting mode at ≈ 5 × 10^4^ cm^−3^.

Experimental parameters and data acquisition were controlled by custom software written in our laboratory. *D*_m_ (15 samples equally log spaced spanning 50 nm ≤ *D*_m_ ≤ 825 nm) was set by a computer and the particle counts were allowed to stabilize for 30 s. The *N*_U_ and *N_D_* values were then recorded for 30 s at 1 Hz, after which the next *D*_m_ was sequentially selected. The 30 s data collection (representing one technical replicate) was then averaged, and a 1*σ* standard deviation was calculated. The *FE* was then calculated from Eq. (1). The total time required to collect an *FE* curve for a single fabric sample was ≈ 15 min.

For all *FE* values, an uncertainty of 5% was assumed, which was derived from: (1) measured 1*σ* standard deviations of *N* ≈ 2.5%, (2) CPC accuracy ≈ 3% (we assumed this was constant for all *N* and not just *N* < 2 × 10^4^ cm^−3^), (3) an ≈ 2% day-to-day variability in *FE* for the same sample, and (4) an ≈ 1% sample-to-sample variability in *FE* for the same material. Samples used for filtration measurements were cut into a circular shape of 2.5 cm diameter using a cleaned stainless-steel form and scissors. Samples were used as received and were equilibrated at ≈ 22 °C. The measured samples did not shed particles under the flow conditions described in this study. Three replicates were cut and were measured from each sample. The data from each replicate were averaged and reported. Uncertainty in *FE* was 5% due to variability in air flow through the filter and counting statistics from both particle counters.

**Fig. 20:**
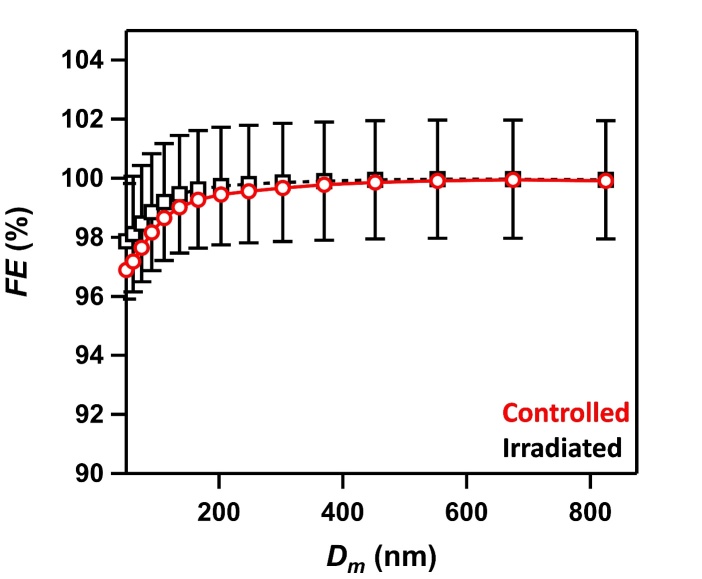
Filtration efficiency (*FE,* %) as a function of particle mobility diameter (*D*_m_) for selected control samples (non-irradiated) (red squares) and UV-C irradiated samples (black squares). Uncertainties (1σ) in *FE* are ± 5% and are only shown for the irradiated sample for clarity. Lines are shown to guide the eye.

Filtration efficiencies were measured as a function of particle size for both control and irradiated N95 samples, and selected results are shown in Fig. 20. The measured *FE* was the lowest (≈ 97%) at 50 nm *D*_m_, due to the dependence of particle diffusion on size. For particles > 150 nm, the FE was > 99% for both the control and irradiated samples. The data shown in Fig. 20 show no significant change in the measured *FE* due to sample irradiation. The difference in *FE* was < 0.5% between control and irradiated samples at each particle diameter across all measured particle diameters, which is an order of magnitude smaller than the type B uncertainty (5%).

### 2.7 Additional Filter Efficiency, Fit, and Strap Tensile Strength Measurements (NPPTL)

**Fig. 21:**
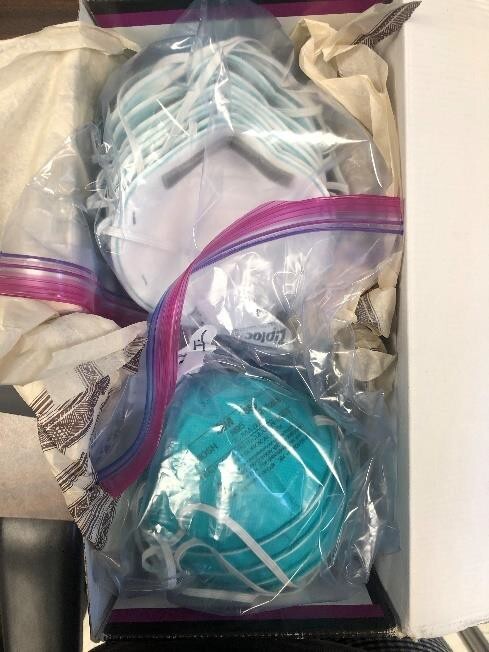
Samples received by NPPTL from NIST. Photo credit: NPPTL

The NPPTL tested ten 3M Model 1860 masks as part of this project, seven that were subjected to 10 cycles of UV-C irradiation and three that were untreated controls, as described in Sec. 2.1 [45]. NIST sent the samples to NPPTL by mail (see Sec. 2.1). NPPTL received the samples as in Fig. 21.

The complete NPPTL test report is available [45], and a summary is given here. All respirators had filter efficiency greater than 95%, showed passing manikin fit factors, and showed up to 4% decrease in the force from the top and bottom straps.

**Fig. 22:**
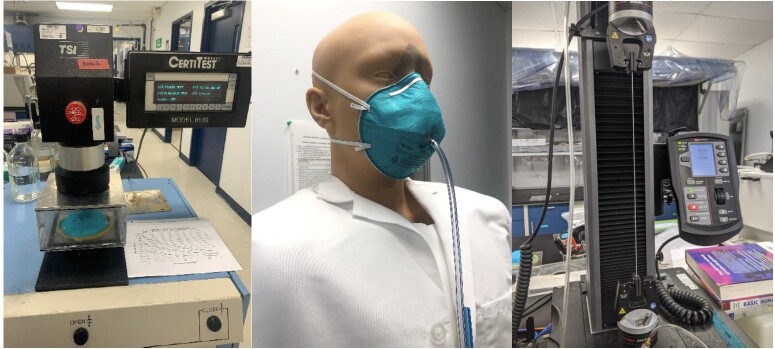
Left: TSI 8130 Filter Tester for particle filter efficiency evaluation (see Table 2). Middle: Static advanced headform used for the manikin fit evaluation (see Table 3). Right: Instron 5943 Tensile Tester used for tensile testing of straps (see Table 4). Photo credits: NPPTL

Per Ref. [45]: “The samples were tested using a modified version of the NIOSH Standard Test Procedure (STP) TEB-APR-STP-0059 to determine particulate filtration efficiency. The TSI, Inc., model 8130 using sodium chloride aerosol was used for the filtration evaluation. For the laboratory fit evaluation, a static manikin headform was used to quantify changes in manikin fit factor. The TSI, Inc., PortaCount® PRO+ 8038 in ‘N95 Enabled’ mode was used for this evaluation. Additionally, tensile strength testing of the straps was performed to determine changes in strap integrity. The Instron® 5943 Tensile Tester was used for this evaluation.” See Tables 2–4 and Fig. 22.

**Table 2:** Particle filter efficiency evaluation [45].^a^

Respirator Model, Decontamination Method, and Number of Cycles	Treated Sample Number	Flow Rate (L min^−1^)	Initial Filter Resistance (mm H_2_O)^b^	Initial Percent Leakage (%)	Maximum Percent Leakage (%)	Filter Efficiency (%)
3M 1860, controls^c^	Control 1	85	9.1	0.390	0.654	99.35
	Control 2	85	9.0	1.42	2.20	97.80
3M 1860, UV-C (1000 mJ/cm^2^), 10 cycles^c^ Minimum filter efficiency 98.97% Maximum filter efficiency 99.61%	1	85	9.4	0.762	1.03	98.97
	2	85	10.0	0.311	0.465	99.54
	3	85	9.4	0.326	0.529	99.47
	4	85	9.7	0.168	0.388	99.61
	5	85	10.0	0.944	0.944	99.06

a Notes: The test method utilized in this assessment was not the NIOSH standard test procedure that is used for certification of respirators. Respirators assessed to this modified test plan do not necessarily meet the requirements of STP- 0059, and therefore cannot be considered equivalent to N95 respirators that were tested to STP-0059.

b mm H_2_O is the unit of measure reported by the NPPTL. The SI unit is the pascal (Pa).

c Controls were non-irradiated N95 FFRs and samples were N95 FFRs irradiated in the UV-C enclosure. None of these samples contained microorganisms. See Sec. 2.1 for more information on the samples, the UV-C enclosure, and the irradiation of the samples.

**Table 3:** Manikin fit evaluation [45].^a^

ManikinFit Factor (MFF) of Decontaminated N95s					
Respirator Model, Decontamination Method, and Number of Cycles	Treated Sample Number	mFF Normal Breathing 1	mFF Deep Breathing	mFF Normal Breathing 2	Overall Manikin Fit Factor
static advanced medium headform (Hanson Robotics)	control 3	127	99	123	115
3M 1860, UV-C (1000 mJ/cm^2^), 10 cycles^b^ static advanced medium headform (Hanson Robotics)	6	200+	200+	200+	200+
	7	200+	101	158	141

a Notes: Per the U.S. Occupational Safety and Health Administration (OSHA) Regulation 1910.134(f)(7)^19^, if the fit factor as determined through an OSHA-accepted quantitative fit testing protocol is equal to or greater than 100 for tight-fitting half facepieces, then the fit test has been passed for that respirator. This assessment does not include fit testing of people; it is a laboratory evaluation that only uses two exercises (normal and deep breathing) on a stationary (nonmoving) manikin headform, and so it varies greatly from the OSHA individual fit test. Therefore, fit results from this assessment cannot be directly translated to results from the standard OSHA-accepted test. Instead, this testing provides an indication of the change in fit performance (if any) associated with the decontamination of respirators.

b Controls were non-irradiated N95 FFRs and samples were N95 FFRs irradiated in the UV-C enclosure. None of these samples contained microorganisms. See Sec. 2.1 for more information on the samples, the UV-C enclosure, and the irradiation of the samples.

19 See https://www.osha.gov/laws-regs/regulations/standardnumber/1910/1910.134

**Table 4:** Strap integrity evaluation [45].

Tensile Force in Respirator Straps of Decontaminated N95s (Recorded Force Values Are at 150% Strain)			
Respirator Model, Decontamination Method, and Number of Cycles	Straps from Treated Sample Number	Force in Top Strap (N)	Force in Bottom Strap (N)
3M 1860, controls^a^	Control 1	3.074	2.749
	Control 2	2.408	2.927
	Control Strap Average:	2.741	2.838
3M 1860, UV-C (1000 mJ/cm^2^), 10 cycles^a^	1	2.775	2.726
	2	2.711	2.608
	3	2.647	2.842
	Decontaminated Strap Average:	2.711	2.725
	% Change ((Decontaminated – Controls)/ Controls):	-1.09%	-3.98%

a Controls were non-irradiated N95 FFRs and samples were N95 FFRs irradiated in the UV-C enclosure. None of these samples contained microorganisms. See Sec. 2.1 for more information on the samples, the UV-C enclosure, and the irradiation of the samples

In summary, for the filtration efficiency results, all N95 FFRs measured greater than 95% filtration efficiency (Table 2). The manikin fit factor results showed passing fit factors (greater than 100) for all N95 FFRs evaluated (Table 3). The strap integrity results showed no visual degradation of the straps; a decrease in recorded force for both the top and bottom straps were measured and showed up to 4% decrease (Table 4).

## 3 Summary

The collaborative effort among UV-Concepts, ResInnova Laboratories, NIST and NPPTL enabled a multi-step, detailed metrology investigation to evaluate the application of a UV-C enclosure for the disinfection and reuse of N95 FFRs. The operational testing by UV-C Concepts and ResInnova Laboratories, with input from NIST on the dose and UV sensor calibrations, revealed that the optical radiation was calibrated and distributed effectively on the irradiated N95 FFRs. The effectiveness of the irradiation was confirmed by the ResInnova Laboratories virology measurements indicating up to a 100% viral reduction relative to the controls. SEM, flow resistance, tensile strength, and particle filtration metrology by NIST provided results to support the conclusion that the UV-C irradiation had no significant effect on the physical characteristics of the N95 FFRs. Testing by the NPPTL for filter efficiency, fit, and strap tensile strength provided results that were consistent with the NIST results and our conclusion. These data and conclusions are consistent with other evaluations of UV-C irradiation on N95 FFRs [13, 46]

Our observations on the performance of the UV-C enclosure are also consistent with a study performed by the University of California Los Angles hospital [47], UCLA Health. A modus operandi for the UV-C enclosure was developed and implemented to meet the high demand for N95 FFRs in response to the onset of COVID-19 inpatients. This led to a decrease in the consumed N95 FFR rate to < 40,000 per month for the hospital system, down from > 83,000 per month the month prior to the implementation of the modus operandi, cutting the demand by nearly half. UCLA Health concluded the process was “a safe and effective way to slow the consumption rate of N95 respirators” [47].

In closing, N95 FFRs continue to be in demand, domestically and internationally, due to the COVID- 19 pandemic, and the interest in using UV-C radiation for disinfection and reuse of N95 FFRs remains high [48]. There has been a considerable increase in demand for N95 FFRs in response to the Delta and Omicron variants of SARS-CoV-2 [49], especially given the increased risks of infectivity associated with their aerosol transmission [50]. In addition, everyday care around the world involving other transmissible diseases, such as measles, continues to draw on the global supply of N95 FFRs. Reuse is also of interest to reduce waste and environmental pollution [51]. Our data, in combination with other studies, will be useful for the development and implementation of N95 FFR disinfection and reuse strategies. These strategies are especially important to support N95 FFR contingency planning (expected shortages), and crisis planning (known shortages).

This article is not intended to quantify the safety of any N95 FFR. It is highly recommended that testing should be undertaken on any products or systems being considered for N95 FFRs or any PPE disinfection and reuse. Previous studies on UV disinfection of N95 FFRs for reuse strategies clearly indicated the importance of measuring the integrity of respirator fit and seal following any disinfection treatment [13, 46]. It is also important to seek guidance from impartial authorities to confirm that any implied standards are met through testing and evaluation. This is especially true for face coverings because these highly differ from one another and require different types of tests and evaluations for their reuse [52].

## 4 Future Studies

Future studies should consider evaluating additional functions of N95 FFRs after being irradiated with UV-C, such as fluid absorption due to breath or phlegm on the interior surface. Effects of soiling due to the use of N95 FFRs, such as from saliva, skin oils, or cosmetics, should also be evaluated. In addition, some commercially available chambers feature motion to enhance disinfection by effectively making the UV-C dosing more uniform and reducing the shadowing and angle of incidence issues raised earlier in this article. There are several ways to actuate this on an N95 FFR or a rack of N95 FFRs, but this requires future study from the point of view of the ray tracing profiling as described in Sec. 2.2.

Last, this work examined disinfection of a surface that was textured, soft, and porous, but these parameters were not specifically investigated here. These are important to consider in future metrology studies, especially from a regulatory vantage point. For example, the U.S. Environmental Protection Agency approves chemical disinfectants only for hard nonporous surfaces, and their test protocols are performed on non-textured surfaces. This represents an advantage of UV-C irradiation over chemicals for disinfection of masks. Moreover, the data presented in this work suggest that the approach used here could have applications for other surfaces such as blood pressure cuffs, fabrics, vinyls, and hospital privacy curtains, to name a few examples, and these should be considered for future studies [53].

## Supplemental Materials

• Supplemental video: http://www.kaltura.com/tiny/0lqfw
  o Title: “With UV Light, N95 Masks Can Be Cleaned and Reused Safely”
  o Description: Researchers at NIST have discovered that, under specific conditions, UV can adequately disinfect masks without causing any unwanted alterations. The findings mark a key step towards the development of standard, science-backed UV disinfection methods that could be critical in the future if the PPE supply is low.
